# Is Dental Amalgam a Higher Risk Factor rather than Resin-Based Restorations for Systemic Conditions? A Systematic Review

**DOI:** 10.3390/ma14081980

**Published:** 2021-04-15

**Authors:** Gianni Gallusi, Antonio Libonati, Mario Piro, Virginia Di Taranto, Edoardo Montemurro, Vincenzo Campanella

**Affiliations:** Department of Clinical Sciences and Translational Medicine, University of Rome “Tor Vergata”, Viale Oxford, 81, 00133 Rome, Italy; gianni.gallusi@gmail.com (G.G.); antlib76@libero.it (A.L.); mario.piro1990@libero.it (M.P.); edoardo.montemurro@gmail.com (E.M.); vincenzo.campanella@uniroma2.it (V.C.)

**Keywords:** dental amalgam, disease, dental restoration, mercury, systematic review

## Abstract

Objective: The aim of this study was to confirm the hypothesis that patients with one or more amalgam restorations have an increased risk for systemic diseases rather than patients with resin-based restorations. Data: The data search produced an initial 3568 total number of records. All titles and abstract were reviewed by five independent examiners, and only 36 records were selected for full text in depth examination. Out of these, only nine publications matched the inclusion criteria and were included in this systematic review. Sources: Electronic databases (MEDLINE, Scopus, Embase, and Web of Knowledge) were searched up to June 2019. In addition, a manual search was carried out on journals related to this topic. Study selection: All selected human clinical studies compared patients with dental amalgam restorations to patients with non-amalgam restorations on restorative material related diseases/health conditions with at least 50 patients and a reasonable follow up. The systemic effects of dental restorations were analyzed. As for any systemic effects, there was no difference between amalgam and composite restoration. Conclusions: With the limitations of the few available randomized controlled trials (RCTs) on the matter, amalgam restorations, similarly to other modern resin-based materials, were not related to an increased risk of systemic diseases or conditions. Clinical significance: On the basis of the available RCTs, amalgam restorations, if compared with resin-based fillings, do not show an increased risk for systemic diseases. There is still insufficient evidence to exclude or demonstrate any direct influence on general health. The removal of old amalgam restorations and their substitution with more modern adhesive restorations should be performed only when clinically necessary and not just for material concerns. In order to better evaluate the safety of dental amalgam compared to other more modern restorative materials, further RCTs that consider important parameters such as long and uniform follow up periods, number of restorations per patient, and sample populations representative of chronic or degenerative diseases are needed.

## 1. Introduction

Dental amalgam has been one of the most used restorative materials for a century and a half.

The use of this material is still widespread on a global level (especially in situations that present a higher risk of caries) because its use entails a series of advantages. Some of the main ones are its excellent mechanical properties (malleability, strength, and ease of application) and durability [1,2,3].

As a result, it is particularly indicated in areas exposed to large mechanical stresses and where a high level of aesthetics is not required such as posterior areas, large carious cavities, and patients with poor oral hygiene [4]. Other reported advantages are low cost, ease of use, longevity, less sensitivity to clinical techniques, and bacteriostatic effect [5,6,7,8].

For these reasons, since its introduction, generations of patients have been treated with amalgam restorations realized according to the classic macro-retentive principles of G.V. Black [9].

Dental amalgam is a solid emulsion made up of two main components: mercury (50%) and alloy powder (50%), composed of silver, tin, copper and other metals (<3%) [10,11].

Due to its mercury content, poor aesthetics, and environmental pollution (due to its disposal and storage), the use of amalgam in dentistry has been the subject of repeated controversy [12].

The most important controversy was about its potential negative effects on general health of patients [13,14,15]. The first concerns were born in the year 1843, when the American Society of Dental Surgeons (ASDS), founded in New York City, affirmed that amalgam restorations are dangerous both for patients and dentists because amalgam causes mercury toxicity, with potential health risks.

Thus, ASDS suggested that all members not use amalgam [16].

In the first decades of the last century (between the 1920s and 1930s), it was understood how much mercury was harmful to human health.

In the 1970s, the presence of mercury was identified in tissues of the body, likely because of the use of dental amalgam. In 1978, Craelius demonstrated a correlation between multiple sclerosis and dental caries likely related to the presence of mercury in amalgam restorations [17,18].

These statements on the potential dental amalgam toxicity were confuted in 1991 when the National Institute of Health–National Institute for Dental Research (NIH–NIDR) and the FDA declared that there was no scientific evidence demonstrating the harmfulness of dental amalgam to patient health [11,19].

However, in 1992, the German health authorities issued the following recommendations to limit the use of amalgam fillings: “There is no scientific proof that placing, removing, or having amalgam restorations in the mouth contains any health risk. However, it is recommended for preventive reasons to restrict its use” [20].

In order to understand how amalgam releases mercury with possible harmful effects on the health of the patient and dentist, it is necessary to understand the different forms of mercury. Mercury has three different forms: elementary or metallic, inorganic, and organic.

The exposure of the population mainly concerns organic methylmercury and is due to the consumption of contaminated fish, while part of the exposure concerns inorganic and elemental mercury and is probably related to the procedures concerning the use of amalgam fillings [21,22,23,24].

Mercury exposure due to amalgam restorations is mainly through the inhalation of vapors generated by the fillings, which enter the circulatory system and reach body tissues such as the central nervous system and the kidneys, and the mercury remains there for long periods of time [25,26,27]. According to an experimental study, the principal route that mercury accesses in the human body derives from the formation of mercury vapors, which can be inhaled and absorbed through the lungs with an efficiency of 80%, while the absorption of metallic mercury through the skin or gastrointestinal tract has proved to be of little importance [28].

Exposure to mercury from dental amalgam mainly occurs during the procedures for positioning and removing the amalgam filling.

Once the hardening reaction is complete, less mercury (well below the current standard limit of what is considered harmful) is released [29,30].

The exposure to mercury from a restoration depends on different variables such as the number and size of restorations, the composition of dental amalgam, the brushing of the teeth, and chewing habits.

In the 2015 Scientific Committee on Health and Environmental Risks (SCHER) opinion on the environmental risks and indirect health effects of mercury from dental amalgam, on the basis of the available data, it was stated that it was not possible to carry out a precise scientific assessment of the real environmental and human health risks related to mercury from the use of dental amalgam because only a preliminary risk assessment could be performed [31].

Many different EU countries are currently phasing down the use of amalgam, but there has still not been a systematic review that shows whether dental amalgam restorations represent a real health risk for patients [32].

Therefore, the aim of this systematic review was to confirm the hypothesis: “patients with one or more amalgam restorations have an increased risk for systemic diseases rather than patients with resin-based restorations.”

## 2. Materials and Methods

### 2.1. Protocol Development and Eligibility Criteria

This systematic review was carried out following guidelines from the Cochrane Handbook for Systematic Reviews of Interventions [33] and PRISMA (Preferred Reporting Items for Systematic Reviews and Meta-Analyses) [34].

The methodology follows the PICOS (Population, Intervention, Comparison, Outcome and Studies) format:

- (P): Patients with at least one permanent posterior tooth requiring a restoration with direct filling material.
- (I): The use of amalgam as dental restorative material.
- (C): All possible comparisons among amalgam restorations and non-amalgam restorations (resin-based composite material or compomer restoration).
- (O): Type of outcome measures (all diseases and/or conditions probably related to the use of dental amalgam).
- (S): Type of studies. Only Randomized Controlled Trials (RCTs) with at least 21 days follow-up and including at least 50 patients for each arm.

### 2.2. Information Sources and Search (Search Strategy)

An electronic bibliographic search was carried out on three databases (MEDLINE, EMBASE, and Scopus) and was carried out by two authors (M.P. and A.L.) who independently selected each other all the articles relating to dental amalgam.

The search strategy was as follows:

(Dentistry) AND ((Restoration) OR (Dental Restoration) OR (composite resins) OR (Composite dental resin) OR (resin-based composites) OR (glass ionomer cements) OR (Amalgam) OR (Dental amalgam)) AND ((Mercury) OR (Metallic mercury) OR (Inorganic Mercury) OR (Methylmercury) OR (Ethylmercury) OR (Phenylmercury) OR (Mercury vapor) OR (Mercury toxicokinetic) OR (Mercury toxicodynamic) OR (Mercury levels)) AND ((Health complaints) OR (Health) OR (health seeking) OR (illness experiences) OR (Health effects) OR (Health complaints) OR (Symptoms) OR (disorders) OR (diseases) OR (syndrome) OR (Asperger’s syndrome) OR (autism) OR (Hypersensitivity) OR (allergy) OR (systemic health effects) OR (physical disorders) OR (psychological disorders) OR (Multiple sclerosis) OR (Amyotrophic lateral sclerosis) OR (Alzheimer’s disease) OR (Parkinson’s disease) OR (neurodevelopment) OR (Child Development) OR (Neuropsychological tests) OR (developmental delay) OR (neurodevelopmental disorder) OR (neurodevelopment)).

A manual search was also carried out limited only to the articles published between January 2001 and June 2019 in the following journals: *Journal of Dental Research*; *Dental Materials*; *Oral Oncology*; *Journal of Endodontics*; *International Endodontic Journal*; *Molecular Oral Microbiology*; *Journal of Dentistry*; *International Journal of Oral Science*; *Oral Diseases*; *Clinical Oral Investigations*; *Caries Research*; *Community Dentistry and Oral Epidemiology*; *Journal of the American Dental Association*; *Journal of Oral Pathology & Medicine*; *Archives of Oral Biology*; *Journal of Orofacial Pain*; *Operative Dentistry*; *Journal of Public Health Dentistry*; *Odontology*; *European Journals of Oral Sciences*; *International Journal of Pediatric Dentistry*; *International Dental Journal*; *BMC Oral Health*; *The European Journal of Prosthodontics and Restorative Dentistry*; *Dental Materials Journal*; *Journal of Applied Oral Science*; *Journal of Oral Science*; *American Journal of Dentistry*; *Journal of Esthetic and Restorative Dentistry*; *Community Dental Health*; *Journal of Dental Sciences*, *Oral Health & Preventive Dentistry*; *European Journal of Paediatric Dentistry*; and *Journal of Clinical Pediatric Dentistry*.

An update of the search was attempted in March 2021 but provided no new articles eligible for the systematic review.

Furthermore, through the bibliography of the identified articles, further research was carried out on any studies published in journals other than those that were manually identified.

### 2.3. Study Selection

The authors designed the protocol following the PRISMA statement. In accordance with the PICOS format, a specific working hypothesis was formulated: “patients with one or more amalgam restorations have an increased risk for systemic diseases rather than patients with resin-based restorations.”

Inclusion Criteria:

For the study selection, the inclusion criteria were as follows:

- Publication in peer-reviewed literature.
- Only human clinical studies, designed as RCTs, that evaluate if patients with amalgam restorations have an increased risk for diseases and/or systemic conditions compared to patients with non-amalgam restorations.
- An observation period after restoration of at least 21 days.
- Full text written in English language.

Exclusion Criteria:

- Full text written in languages different from English.
- Animal studies, in vitro experiments, clinical studies on dental amalgam without a control group, and articles that do not present important data such as the number of patients.
- Case reports, expert opinions, and narrative reviews on dental amalgam.
- Articles not providing data on the number of patients included in the study, number of amalgam restorations and/or the total number of surfaces restored with amalgam, and follow-up time.

The data extraction from each RCT included in this review was carried out following the PICOS criteria and using two tables.

In Table 1, the following information is shown:

- Country in which the study was conducted (USA, Turkey, Portugal).
- Number (n°) of centers (single-center or multicentric).
- Follow-up of each study (7 years, 5 years, and 21 days).
- The type of Interventions (dental amalgam compared to resin-based composite material).
- The type of participants (the number of randomized patients compared to number of analyzed patients).
- The type of systemic conditions and the secondary outcomes probably related to the amalgam or resin-based material.

In the other table (Table 2), the follow information is shown:

- Journal.
- Year of publication.
- Number of patients in test group and in the control group.
- Cumulative number of surfaces restored with amalgam.
- Mean surface restored with other materials.
- Disease incidence related to amalgam or resin-based material.

All studies that met the inclusion criteria were included and subjected to quality assessment and data extraction.

In the first step, titles and abstracts (when available) of all the articles identified through electronic searches were reviewed for eligibility. This was done individually by the three authors (G.G., V.D.T., and E.M.). This comprised the 1st screening.

Subsequently, for all studies that met the inclusion criteria and for all those that were in doubt due to lack of data from the title or the abstract, the full text was assessed in order to establish whether the studies actually met the established inclusion criteria.

In the first screening, 36 papers passed. For all papers, full texts were collected and submitted to two review authors (M.P. and A.L.) for in depth analysis. Any inconsistencies were discussed by the two review authors and submitted to a third review author (V. C.) to resolve. Out of the 36 papers, 27 were excluded for not matching the inclusion criteria. All reasons for studies exclusion were recorded and are reported in Table 3.

The risk of bias was also recorded.

This procedure was carried out by five investigators (G.G., A.L., M.P., V.D.T., and E.M.) through independent research. Any doubts or inconsistencies between these searches were addressed with the consultation of a final reviewer (V.C.). Any missing data or necessary clarifications were requested directly from the author of each RCT.

### 2.4. Data Items

Primary and secondary outcomes were assessed.

Primary outcome measures included the rate of all diseases and/or conditions probably related to the use of dental amalgam.

Secondary outcomes measures included the number of restorations, type of restorations, and disease/condition’s onset period.

### 2.5. Quality Assessment

Data extraction (patients, restorations, and conditions or diseases likely related to the amalgam) was completed using a specific methodology developed for subsequent steps, which led to preliminary testing on other studies and then adjusting as needed.

During the data extraction process, the quality and risk of bias assessments were also performed using the Cochrane collaboration’s tool for assessing risk of bias [34].

This was done independently by three authors (G.G., A.L. and M.P.), who discussed any discrepancies to reach a shared consensus. When this was not possible, a fourth author (V.C.) was consulted.

**Figure 1:**
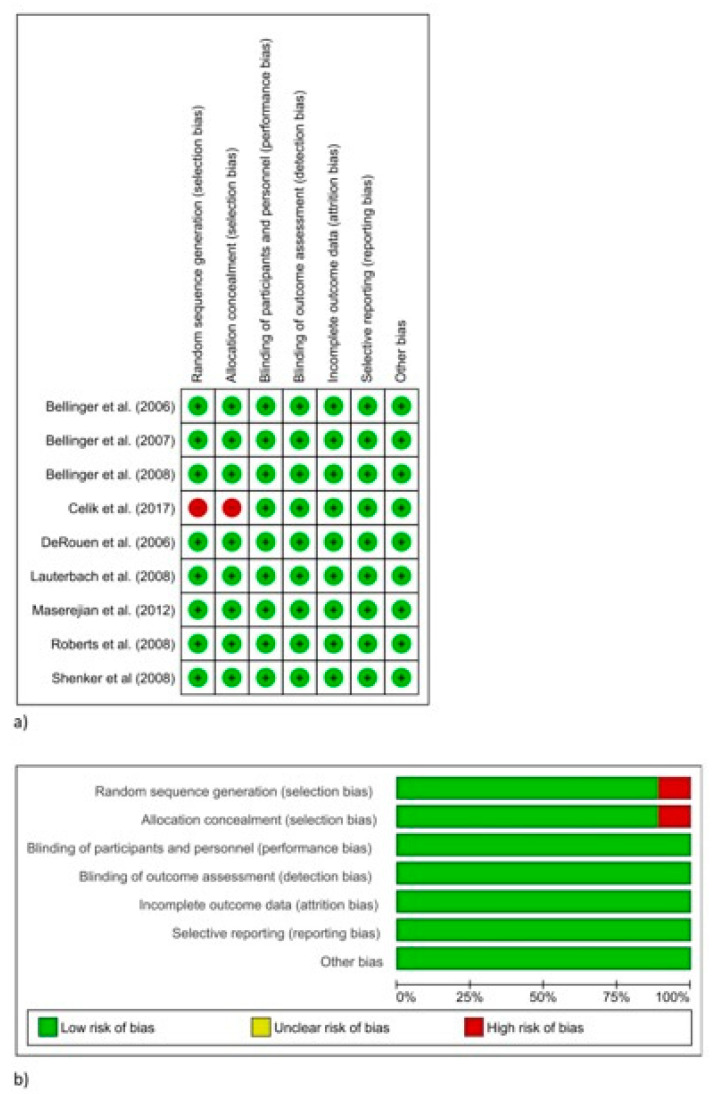
Risk of bias summary of the included studies using the Cochrane collaboration’s tool [35]. (**a**) Presented for each included study. (**b**) Presented as percentages across all included studies. A green circle/area indicates a low risk of bias for the study regarding the specific domain, a red circle/area indicates a high risk of bias, and a yellow circle/area indicates that the risk is unclear.

The risk of bias assessment is reported in Figure 1. This evaluation was done using the Cochrane collaboration’s tool for assessing the risk of bias [35].

### 2.6. Data Synthesis

In order to highlight the collected data and analyze the variations of the study with regard to results and peculiarities, the data are presented in explanatory tables followed by a descriptive summary. This allowed us to highlight similarities and discrepancies and facilitated the possibility of further synthesis or the application of other comparison methods.

## 3. Results

### 3.1. Study Selection

**Figure 2:**
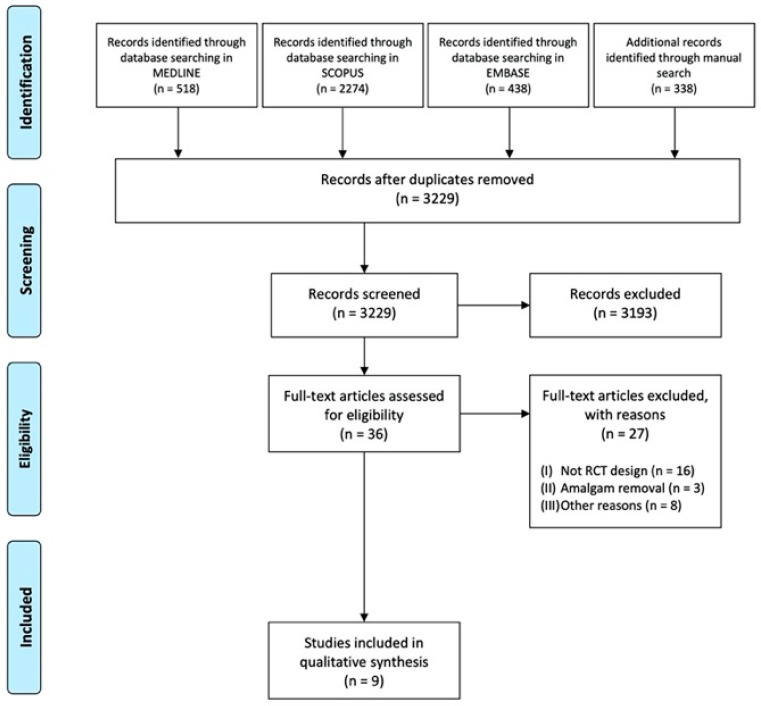
Preferred Reporting Items for Systematic Reviews and Meta-Analyses (PRISMA) 2009 flow diagram for study selection [34].

The flow diagram of the study selection process is shown in Figure 2.

The electronic search in the databases of MEDLINE, Scopus, and EMBASE found 3230 studies (518 (MEDLINE), 2274 (Scopus), 438 (EMBASE)), and the manual search provided 338 additional publications.

After the elimination of duplicates, 3229 were the remaining articles.

After the abstract and title examination, 3193 articles out of 3229 articles initially found were excluded.

The full text of the 36 remaining articles was downloaded and observed in detail.

Of these 36 articles, 27 were discarded because they did not meet the established inclusion criteria (Table 3 shows the articles with the full text excluded and the reasons for exclusion).

Consequently, the total number of RCTs that met the inclusion criteria and that were included in this study was nine (Figure 2).

### 3.2. Characteristics of the Included Studies

An overview of the nine included RCTs is reported in Table 1. Five of them referred to the New England Children’s Amalgam Trial, a randomized multicentric safety trial of amalgam with five-year follow-up conducted from 1997 to 2006 [36,37,38,39,40].

Three of the included studies referred to the Casa Pia Study of the Health Effects of Dental Amalgams in Children, a randomized clinical trial design to evaluate the safety of low-level mercury exposure from dental amalgam fillings in children with a seven-year follow-up conducted from 1997 to 2005 [41,42,43].

The remaining trial [44] was an RCT that evaluated the changes in the oral environment caused by the different materials used for fifth class restorations measuring gingival crevicular fluid inflammatory cytokine levels.

Five studies were multicenter trials conducted in the USA [36,37,38,39,40].

Three studies were single-center trials conducted in Portugal (Lisbon) [41,42,43].

One study was a single-center trial conducted in Turkey [44].

### 3.3. Characteristics of the Participants

The results of the individual included studies are shown in Table 2.

The total number of participants from the nine RCTs was 3345. Of these 3345, the data of 2884 participants were examined.

The age of the participants varied from six to twelve years at the baseline. The follow-up period instead ranged from 21 days to 7 years. The cumulative number (n°) of surfaces restored with amalgam ranged from 11.5 to 16.1. The mean surface restored with other material ranged from 15.8 to 21.3.

### 3.4. Characteristics of the Interventions

In six RCTs, the “interventions” were amalgam or resin-based composite material restorations [36,39,40,41,42,43]. In two RCTs, the interventions were amalgam, compomer, or composite restorations [37,38]. In only one study, participants received composite resin, compomer resin, glass ionomer cement, or amalgam [44].

### 3.5. Characteristics of Outcomes

The primary outcomes were: IQ scores [36]; Full-Scale IQ score on the Wechsler Intelligence Scale for Children-Third Edition (WISC-III) [37]; the parent-completed Child Behavior Checklist (CBCL) [38]; Plaque index (PI), gingival index (GI), and gingival crevicular fluid (GCF) volume [44]; memory, attention/concentration, and motor/visuomotor; nerve conduction velocity [41]; neurological hard signs (NHSs) and presence of tremors [43]; BMI-for-age, body fat percentage, and height velocity [39]; oral HG-resistant bacteria [42]; and white cell numbers, t-cell, b-cell, neutrophil and monocyte responsiveness [40].

The secondary outcomes were: tests of memory; visuomotor ability; renal glomerular function [36]; General Memory Index (GMI) from the Wide Range Assessment of Memory and Learning and the Visual–Motor Composite (VMC) from the Wide Range Assessment of Visual Motor Abilities [37]; Behavior Assessment System for Children (BASC-SR) [38]; GCF IL-6, IL8, and TNF-α levels [44]; renal glomerular function [41]; neurological soft signs (NSSs) [43]; age of menarche [39]; and urine Hg-resistant bacteria [42]. Only one study did not report secondary outcomes [40].

### 3.6. Excluded Studies

Of 36 text articles assessed for eligibility, 27 studies were excluded for not meeting the inclusions criteria. In particular, the main reasons for exclusion were: text written in languages different from English, animal studies, in vitro experiments, clinical studies on dental amalgam without a control group, and articles that did not present important data such as the number of patients, case reports, expert opinions, and narrative reviews on dental amalgam.

Table 3 shows, in detail the articles with full text excluded and the reasons they were excluded.

## 4. Discussion

### 4.1. Main Results

Based on the available included RCTs, no evidence was found to confirm the hypothesis that patients with one or more amalgam restorations have an increased risk for systemic diseases rather than patients with resin-based restorations.

Long term effects on physical development and neurophysiological, immune, and renal function were investigated as systemic consequences due to mercury absorption [36,37,38,39,40,41,43], while changes in the presence of resistant bacteria were evaluated as local consequences [42,44].

Patients undergoing amalgam restorations showed higher urinary, hair, and blood mercury concentrations, though not enough to cause measurable systemic effects.

All studies compared amalgam to other restorative materials (most commonly resin composites). There were no studies in which patients with amalgam restorations were compared with Caries free patients.

Follow-up period ranged between 21 days and 7 years: it is likely that the follow-up period was too brief for adverse effects from chronic mercury vapor release to be revealed. Generally, an amalgam restoration, being very long-lasting, remains in place for many years and thus releases mercury for a considerably longer period of time.

Furthermore, the minimum age patient age is six years old, so the results of these studies may not concern children under the age of six, where the sensitivity to mercury toxicity may be greater.

### 4.2. Interpretation in the Context of the Available Literature

Bellinger DC. et al. (2006) (New England Children Amalgam Trial: NECAT) [36] evaluated the neuropsychological and renal function in children treated with amalgam or composite restorations after a five-year follow-up period.

The primary neurological outcome was five-year change in Full-Scale IQ scores on the Wechsler Intelligence Scale for Children-Third Edition (WISC-III).

This parameter was measured at baseline, three years, and five years after initial treatment.

During the IQ test, the Wechsler Individual Achievement Test and Behavior Assessment System for Children were also performed at each visit.

Secondary neuropsychological end points were the GMI and VMC, attention, and emotional state.

Glomerular renal function was assessed by the excretion of albumin, measured in milligrams per gram of creatinine.

The study reported no statistically significant difference in negative neuropsychological and renal activity after five years of follow-up between the amalgam group and the composite group. As a result, the effects of dental amalgam on health cannot be considered as a crucial factor in the choice of a restorative material, but even the elimination of amalgam (very advantageous in some cases) in favor of a composite material can be counterproductive for the oral health of a patient [36].

Bellinger DC. et al. (2007) (New England Children Amalgam Trial: NECAT) [37] further analyzed the neuropsychological (WISC-III Full-Scale IQ, GMI, or VMC) and renal outcomes evaluated by Bellinger et al. (2006) in a more sensitive way.

In the study of Bellinger et al., 2006 [36], mercury exposure (from amalgam restorations) was dichotomously assessed according to the reference group (amalgam treatment group vs. composite group). The limit of this study was that the different dental needs of children and, therefore, the different amounts of exposure to amalgam were not considered.

In the study of Bellinger et al., 2007 [37], instead, these outcomes were continuously analyzed on the basis of two indices: the surface-years of amalgam index (which gives information on the number of dental surfaces restored with amalgam and on the duration of each in years) and the urinary mercury excretion index, a biomarker of the absorbed dose that is related to the number of amalgam restorations.

The results of this study showed that amalgam restorations do not have negative neuropsychological effects in children. This study only considered children who were at least six years old, meaning it was not possible to ascertain whether the vulnerability to mercury varies according to the stage of development. Furthermore, in all likelihood, the sensitivity to mercury could also vary based on the patient’s genotype [37].

Bellinger AD. et al. (2008) (New England Children Amalgam Trial: NECAT) [38]: this study investigated whether, after five years of follow-up, the psychosocial health of dental amalgam-exposed children was worse than that of children treated with amalgam-free restorations (composite resin or compomer). The hypothesis was that the use of dental amalgam would be associated with a worse psychosocial state in children.

The primary outcomes were CBCL scores. The CBCL were completed by a parent before treatment as a baseline and at the end of the study after five years of follow-up.

Secondary outcomes came from the children’s self-reports using the BASC-SR at the end of follow up period of five years.

The psychosocial status of the children was put in relation to mercury exposure by treatment assignment, surface-years of amalgam, and urinary mercury excretion.

This study reported that the psychological health of children in the amalgam group was not affected by amalgam exposure, being in some ways even better than that in non-amalgam group [38].

Results were consistent with previous reports [36] for the primary outcomes of NECAT, but with the same limitations of the previous study.

Celik N. et al. (2017) [44] evaluated the biological effect on the gingival tissue of some materials used in fifth-class restorations, such as composite resins, compomers, amalgam, and ionomer glass.

The hypothesis of this study was that substances released by these materials could be cytotoxic, causing the secretion by these cells of cytokines that trigger the inflammatory process of the marginal gingiva.

Consequently, in order to evaluate the pro-inflammatory effect of these restorative materials, the levels of cytokines, such as IL-6, IL-8, and TNF-α (which are the main mediators of inflammation) in the GCF were detected.

The detections were performed at 7 and 21 days of follow-up.

The results of this study revealed an increase in the levels of cytokines in the GCF, so the surface properties, composition, and cytotoxicity of these materials have a pro-inflammatory effect on the gingival tissue and therefore play fundamental roles in producing changes in the oral environment [44].

De Rouen TA. et al. (2006) (Casa Pia) [41]: this was a randomized clinical trial that also evaluated the effect of dental amalgam on the neurological, neurobehavioral, and renal function of children exposed to mercury from dental amalgam.

This study hypothesized that children exposed to low levels of mercury from amalgam restorations would present worse neurological development and renal health than children who received similar conservative treatments with resin-based restorative materials.

As a result, the neurological and kidney conditions of the children who received amalgam restorations were compared with those of the children who received composite fillings.

The primary outcomes were memory, attention/concentration, motor/visuomotor domain, and nerve conduction velocity.

The secondary outcome was glomerular renal function.

The results of this study confirmed that dental amalgam does not cause harmful neuropsychological and renal effects. Dental amalgam may be a valid treatment option in some cases, but, since it exposes patients to low levels of mercury, it is preferable not to use it [41].

These results were consistent with those of the Bellinger et al., 2006 [36] and Bellinger et al., 2008 [38].

Lauterbach M. et al. (2014) (Casa Pia) [43]: this study focused on the effects of dental amalgam on the neurological and neuro-behavioral state of children exposed to amalgam restorations.

In order to evaluate any differences in effects on the integrity of the central nervous system between children of the amalgam group and those of the non-amalgam group, neurological tests were carried out to detect the presence/absence of NHSs, tremors, and/or mild NSSs.

This study also corroborated the finding that dental amalgam has a completely irrelevant effect on the neurological status of children [43].

Maserejian NN. et al. (2012) (New England Children Amalgam Trial: NECAT) [39] also showed that the presence of composite resin or amalgam restorations does not adversely affect the child’s physical development, including changes in BMI, the percentage of fat mass, and the speed of growth in height [39].

Roberts MC. et al. (2008) (Casa Pia) [42] highlighted that the treatment of carious lesions with dental amalgam does not cause an increase in the levels of oral and urinary bacteria resistant to mercury and the antibiotic, a phenomenon that would be very dangerous in case of bacterial infections.

In addition, there is no scientific correlation between the acquisition of antibiotic and mercury resistance in bacteria [42].

Shenker BJ. et al. (2013) [40]: this was a sub study of the NECAT, in which sample cells taken from the tested children were studied in vitro to evaluate the immunotoxic effects of amalgam restorations.

No significant differences were found in the functionality of the T-cells, B-cells, monocytes, or neutrophils at 6, 12, or 60 months, although at five-to-seven days after treatment, there was a slight and not significant decrease in the activity of T-cells and monocytes.

These results showed that that dental amalgam does not cause relevant alterations of the immune system in children [40].

Data from the included RCTs were not able to highlight any systemic adverse condition related to amalgam fillings, so the removal of old amalgam restorations and their substitution with more modern adhesive restorations should be performed only when clinically necessary and not just for material concerns.

### 4.3. Quality of the Evidence

This systematic review was based on the results of three RCTs that, independently of each other, evaluated whether the presence of dental amalgam represents a more significant risk factor than amalgam-free restorations (composite resin, compomer, or glass ionomer cement): five of the included studies referred to the New England Children’s Amalgam Trial [36,37,38,40,43], three of the included studies referred to the Casa Pia Study of the Health Effects of Dental Amalgams in Children [41,42,43], and the remaining trial [44] was an RCT that evaluated the biological effects of various restoration materials on the composition of the crevicular gingival fluid and therefore the impact they have on the oral environment.

The bias assessment of the included studies is shown in Table 2: eight studies were considered to have a low risk of bias [36,37,38,39,40,41,42,43], while only one study was considered to have a high risk of bias [44].

Two group RCTs only selected children for their possible higher vulnerability to mercury toxicities due to their developmental immaturity and the higher risk of caries treated with amalgam [36,37,38,39,40,41,42,43].

### 4.4. Limitations of the Systematic Review

Only few published RCTs about systemic effects of dental amalgam were found.

Furthermore, these RCTs investigated only a few different conditions, while in the literature, some non-RCT studies have shown that the presence of dental amalgam is likely a major risk factor in reference to some systemic conditions, such as neurodegenerative diseases (Alzheimer disease, multiple sclerosis, etc.).

The NECAT study and the Casa Pia Study only compared restorations in posterior teeth, because all studied anterior teeth were fully restored with composite materials.

Various studies have shown that monomers released from resin materials are harmful to human health. However, these assessments derive mainly from in vitro studies, the results of which could therefore be clearly different from clinical applications.

Furthermore, in eight RCTs, only healthy children were considered. The results of these studies may not be representative for different populations from healthy children such as children or adults with potential mercury-sensitive health conditions, e.g., patients affected by chronic kidney disease.

Children represent the social group that receives most restorations and is exposed to the effects of mercury during their growth and development; therefore, they comprise a good sample for cognitive development assessment. However, to investigate the potentially adverse effects of amalgam restorations, a longer follow-up period should be considered in an attempt to make connections between amalgam exposure and adult or old age health conditions.

## 5. Conclusions

Even after more than one century and a half of clinical use and research, there is still lack of scientific evidence of any systemic adverse effect due to the exposure to amalgam restorations.

From the analysis of the available literature, it was not possible to confirm the hypothesis that “patients with one or more amalgam restorations have an increased risk for systemic diseases rather than patients with resin-based restorations.”

This systematic review highlights the finding that there is no scientific evidence that the presence of dental amalgam increases the risk of contracting systemic diseases.

Even if it is less used in daily practice, amalgam has to be considered a safe and valid restorative material due to its lower failure rate than resin composites. Considering its longevity, the choice of amalgam is actually very cost-effective.

Aesthetic and safety concerns led to the development of mercury-free resin-based restorative materials, but more studies are needed in order to verify their long-term efficacy and general safety.

The results of this systematic review are in accordance with a recent similar review that came to the same conclusions when asking the scientific community for more RCTs on the relationships between dental amalgam, resin-based materials, and health concerns [45].

In order to better evaluate the safety of dental amalgam compared to other, more modern restorative materials, we need further RCTs that consider important parameters such as long and uniform follow up periods, the number of restorations per patient, and sample populations representative of chronic or degenerative diseases.

Further research should also investigate genetic susceptibility to mercury and the potential adverse effects related to exposure to bisphenol A derived from composite materials.

## Figures and Tables

**Table 1:** General overview of the included studies.

		Methods		Interventions		Participants			
Reference	Country	No. Centers	Follow-Up	Test	Control	Randomized	Analyzed	Systemic Condition	Secondary Outcomes
Bellinger DC et al. (2006)	USA	Multi centric	5 years	Dental amalgam	Resin-based composite material	534 (aged 6–10 years at baseline)	449	IQ scores	Tests of memory; visuomotor ability; Renal glomerular function.
Bellinger DC et al. (2007)	USA	Multi centric	5 years	Dental amalgam	Resin-based composite material and compomer	534 (aged 6–10 years at baseline)	434	Full-Scale IQ score on the Wechsler Intelligence Scale for Children-Third Edition (WISC-III)	General Memory Index (GMI) from the Wide Range Assessment of Memory and Learning and the Visual–Motor Composite (VMC) from the Wide Range Assessment of Visual Motor Abilities.
Bellinger DC et al. (2008)	USA	Multi centric	5 years	Dental amalgam	Resin-based composite material and compomer	534 (aged 6–10 years at baseline	395	The parent-completed Child Behavior Checklist (CBCL).	Behavior Assessment System for Children (BASC-SR).
Celik N et al. (2017)	Turkey	Single-center	21 days	Restored sides divided in 4 sub-groups: Group 1: composite resin Group 2: compomer resin Group 3: glass ionomer cement Group 4: amalgam	Healthy sides: Contralateral tooth intact enamel surface	60 (aged 25–40 years)	60	Plaque index (PI), gingival index (GI), and gingival crevicular fluid (GCF) volume	GCF IL-6, IL8, and TNF-α levels
DeRouen TA et al. (2006)	Portugal (Lisbon)	Single-center	7 years	Dental amalgam	Resin-based composite material	507 (aged 8–10 years	507	Memory, attention/concentration and motor/visuomotor nerve conduction velocity	Renal glomerular function
Lauterbach M et al. (2008)	Portugal (Lisbon)	Single-center	7 years	Dental amalgam	Resin-based composite material	506 (aged 8–12 years).	506	Neurological hard signs (NHSs) and presence of tremors	Neurological soft signs (NSSs).
Maserejian NN et al. (2012c Nov)	USA	Multi centric	5 years	Dental amalgam	Resin-based composite material	532 (aged 6–10 years at baseline)	474	BMI-for-age, body fat percentage, and height velocity	Age of menarche
Roberts MC et al. (2008)	Portugal	Single-center	7 years	Dental amalgam	Resin-based composite material	150 children (aged 8–10 years)	150	Oral HG-resistant bacteria	Urine Hg-resistant bacteria
Shenker BJ et al. (2008)	USA	Multi centric	5 years	Dental amalgam	Resin-based composite material	66 (aged 6–10 years at baseline)	59	White cell numbers, t-cell, b-cell, and neutrophil and monocyte responsiveness	Not shown

**Table 2:** Results of the individual included studies in the present systematic review.

Author	Journal	Year	N° Patients Test Group	N° Patients Control Group	Cumulative no of Surfaces Restored with Amalgam	Mean Surface Restored with Other Material	Disease Incidence Amalgam	Disease Incidence Other Materials
Bellinger DC et al.	JAMA	2006	267—baseline 219—5 years	267—baseline 217—5 years	11.5	15.8	No statistical differences.	No statistical differences
Bellinger DC et al.	JADA	2007	267	267	11.5	15.8	Using ANCOVA, we found that none of the three neuropsychological test scores—WISC-III Full-Scale IQ, GMI, and VMC—were associated significantly with either continuously distributed index of mercury dose.	No statistical differences
Bellinger DC et al.	J Dent Res	2008	197	198	11.5	15.8	No statistical differences.	No statistical differences
Celik N et al.	Arch Oral Biol.	2017	60	60	Group 1: 1	Group 2: 1 Group 3: 1 Group 4: 1	After restorative treatments, PI and GI scores were decreased compared with baseline evaluations. There was a significant difference in GCF levels between experimental and control sites in all groups. GCF IL-6 levels in all groups except for Filtek Z250, GCF IL-8 levels in all groups except Fuji IX, and GCF TNF-α levels in only Fuji IX showed significant differences between experimental and control sites.	
DeRouen TA et al.	JAMA	2006	253	254	16.1	21.3	No statistical differences.	No statistical differences
Lauterbach M et al.	JADA	2008	253 at baseline and 136 after 7 years of follow up	253 at baseline and 142 after 7 years of follow up	Not shown	Not shown	No statistical differences.	No statistical differences
Maserejian NN et al.	J Dent Res	2012	266—baseline 238—end	267—baseline 236—end	Not shown	Not shown	No statistical differences.	No statistical differences
Roberts MC et al.	J Dent Res	2008	77 at baseline- 43 at 7 years of follow up	73 at baseline- 38 at 7 years of follow-up	Not shown	Not shown	No statistical differences.	No statistical differences
Shenker BJ et al.	J Am Dent Assoc	2008	35—baseline 29—end	31—baseline 30—end	Not shown	Not shown	No statistical differences.	No statistical differences

**Table 3:** Full-text articles excluded, with reason.

Publication	Reason of Exclusion
Maserejian NN et al. (2012a-August). Dental composite restorations and psychosocial function in children.	The study focused on composite restorations, with no direct comparisons between amalgam restorations and composite restorations.
Maserejian NN et al. (2012b-October). Dental composite restorations and neuropsychological development in children: treatment level analysis from a randomized clinical trial.	The study did not concern amalgam restorations.
Children’s Amalgam Trial Study Group (2003). The children’s amalgam trial: design and methods.	It was a design paper, not an RCT.
Lauterbach M et al. (2010). Randomized Controlled Trial Demonstrates that Exposure to Mercury from Dental Amalgam Does Not Adversely Affect Neurological Development in Children.	It was a review paper, not an RCT.
Richardson GM et al. (2011). Mercury exposure and risks from dental amalgam in the US population, post-2000.	No test group vs control group.
Rothwell JA et al. (2008). Amalgam dental fillings and hearing loss.	No test group vs control group.
Melchart D et al. (2008). Treatment of health complaints attributed to amalgam.	The study concerned amalgam removal.
Lindh Ulf et al. (2002). Removal of dental amalgam and other metal alloys supported by antioxidant therapy alleviates symptoms and improves quality of life in patients with amalgam-associated ill health.	The study concerned amalgam removal.
Gottwald B et al. (2002). Psychological, allergic, and toxicological aspects of patients with amalgam-related complaints.	No test group vs control group.
Aktaş B et al. (2015). The impact of amalgam dental fillings on the frequency of *Helicobacter pylori* infection and *H. pylori* eradication rates in patients treated with concomitant, quadruple, and levofloxacin-based therapies.	It was an RCT, but there was not a clear randomization regarding the rate of *H. pylori* infection according to the presence of amalgam filling.
Geier DA et al. (2012). A dose-dependent relationship between mercury exposure from dental amalgams and urinary mercury levels: a further assessment of the Casa Pia Children’s Dental Amalgam Trial.	No RCT.
Woods JS et al. (2007). The contribution of dental amalgam to urinary mercury excretion in children.	The study concerned the effects of dental amalgam on urinary excretion in children.
DeRouen TA et al. (2002). Issues in design and analysis of a randomized clinical trial to assess the safety of dental amalgam restorations in children.	The study concerned the design and analysis of an RCT on the safety of dental amalgam.
Schuurs A et al. (2000). Biological mercury measurements before and after administration of a chelator (DMPS) and subjective symptoms allegedly due to amalgam.	The study concerned the relationship between subjective symptoms allegedly due to amalgam and mercury measurements before and after the administration of a chelator.
Halbach S et al. (2008). Blood and urine mercury levels in adult amalgam patients of a randomized controlled trial: Interaction of Hg species in erythrocytes.	The study concerned internal exposure to amalgam-related mercury from the kinetics of inorganic Hg in plasma and erythrocytes after amalgam removal.
Sweeney M et al. (2002). The release of mercury from dental amalgam and potential neurotoxicological effects.	It is a vitro study, not an RCT.
Ahlgren C et al. (2014). Contact allergies to potential allergens in patients with oral lichen lesions.	No test group vs control group. Moreover, exclusion criteria included: “Patients with oral mucosal problems or problems allegedly caused by dental materials” and “Patients tested with the dental and cheilitis series.”
Garhammer P et al. (2001). Patients with local adverse effects from dental alloys: frequency, complaints, symptoms, allergy.	No because the selection criteria were intraoral complaints or symptoms, like gingivitis, taste irritation, and dry mouth or burning mouth in relation to metal restorations, except for amalgams.
Joska L et al. (2009). The mechanism of gingiva metallic pigmentations formation.	There was no control group.
Lygre GB et al. (2016). Prenatal exposure to dental amalgam and pregnancy outcome.	No test group vs control group.
Golding J et al. (2016). Dental associations with blood mercury in pregnant women.	No RCT
Lin PY et al. (2018). Risk of subsequent attention-deficit/hyperactivity disorder among children and adolescents with amalgam restorations: A nationwide longitudinal study.	It is a retrospective cohort study, not an RCT.
Lygre GB et al. (2010). Exposure to dental amalgam restorations in pregnant women.	No RCT.
Sundström A et al. (2011). Stressful negative life events and amalgam related complaints.	No RCT.
Naimi-Akbar A et al. (2013). Health related quality of life and symptoms in patients with experiences of health problems related to dental restorative materials.	The study concerned the replacement of dental restorations (amalgam or other materials).
Lygre GB et al. (2003). Reporting on adverse reactions to dental materials. Intraoral observations at a clinical follow-up.	No RCT.
Watson GE et al. (2011). Prenatal exposure to dental amalgam: evidence from the Seychelles Child Development Study main cohort.	No RCT.

## Data Availability

No new data were created or analyzed in this study.

## References

[1] Jones D.W. (1983). The enigma of amalgam in dentistry. J. Canad. Dent. Assoc..

[2] Langan D.C., Fan P.L., Hoos A.A. (1987). The use of mercury in dentistry: A critical review of the recent literature. J. Am. Dent. Assoc..

[3] Mai E.K., Qasem D.A., Ridwaan O. (2014). Factors relating to usage patterns of amalgam and resin composite for posterior restorations—A prospective analysis. J. Dent..

[4] Rathore M., Singh A., Pant V.A. (2012). The dental amalgam toxicity fear: A myth or actuality. Toxicol. Int..

[5] United Nations Environment Programme—Chemicals (2002). Global Mercury Assessment Inter-Organization Programme for the Sound Management of Chemicals.

[6] Cenci M.S., Piva E., Potrich F., Formolo E., Demarco F.F., Powers J.M. (2004). Microleakage in bonded amalgam restorations using different adhesive materials. Braz. Dent. J..

[7] Beyth N., Domb A.J., Weiss E.I. (2007). An in vitro quantitative antibacterial analysis of amalgam and composite resins. J. Dent..

[8] Goldman A., Frencken J.E., De Amorim R.G., Leal S.C. (2018). Replacing amalgam with a high-viscosity glass-ionomer in restoring primary teeth: A cost effectiveness study in Brasilia, Brazil. J. Dent..

[9] Mjör I.A., Gordan V.V. (2002). Failure, repair, refurbishing and longevity of restorations. Oper. Dent..

[10] ISO-Standards ISO 1559 (1995). Dental Materials—Alloys for Dental Amalgam.

[11] Tchounwou P.B., Ayensu W.K., Ninashvili N., Sutton D. (2003). Environmental exposure to mercury and its toxicopathologic implications for public health. Environ. Toxicol..

[12] Dunne S.M., Grainsford I.D., Wilson N.H. (1997). Current materials and techniques for direct restorations in posterior teeth. Part I: Silver amalgam. Int. Dent. J..

[13] (1991). Environmental Health Criteria 118, Inorganic Mercury.

[14] Eley B.M., Cox S.W. (1993). The release, absorption and possible health effects of mercury from dental amalgam: A review of recent findings. Br. Dent. J..

[15] Lorscheider F.L., Vimy M.J., Summers A.O. (1995). Mercury exposure from ‘silver’ tooth fillings: Emerging evidence questions a traditional dental paradigm. FASEB J..

[16] Relyea G.V.N. (1897). Incidents in Office Practice. Am. J. Dent. Sci..

[17] Craelius W. (1978). Comparative epidemiology of multiple sclerosis and dental caries. J. Epidemiol. Community Health.

[18] Aminzadeh K.K., Etminan M. (2007). Dental Amalgam and Multiple Sclerosis: A Systematic Review and Meta-Analysis. J. Public Health Dent..

[19] Mackert J.R., Berglund A. (1997). Mercury exposure from dental amalgam fillings: Absorbed dose and the potential for adverse health effects. Critic. Rev. Oral Biol. Med..

[20] Eley B.M., Cox S.W. (1987). Mercury from dental amalgam fillings in patients. Br. Dent. J..

[21] Langworth S., Elinder G.C., Åkesson A. (1988). Mercury exposure from dental fillings. I. Mercury concentrations in blood and urine. Swed. Dent. J..

[22] Skare I., Engqvist A. (1994). Human exposure to mercury and silver released from dental amalgam restorations. Arch. Environ. Health..

[23] Warfvinge K. (1995). Mercury exposure of a female dentist before pregnancy. Br. Dent. J..

[24] Weiner J.A., Nylander M. (1995). An estimation of the uptake of mercury from amalgam fillings based on urinary excretion of mercury in Swedish subjects. Sci. Total Environ..

[25] Phillips R.W., Avery D.R., Mehra R., Swartz M.L., McCune R.J. (1973). Observations on a composite resin for class II restorations: Tree-year report. J. Prosth. Dent..

[26] Berglund A. (1992). Release of mercury vapor from dental amalgam. Swed. Dent. J. Suppl..

[27] Lygre G., Grønningsæter A., Gjerdet N. (1998). Mercury and dental amalgam fillings. Tidsskr. Nor. Laegeforening.

[28] Arenholt-Bindslev D. (1998). Environmental aspects of dental filling materials. Eur. J. Oral Sci..

[29] European Commission (2001). Ambient Air Pollution by Mercury (Hg).

[30] Barregard L., Trachtenberg F., McKinlay S. (2008). Renal effects of dental amalgam in children: The New England children’s amalgam trial. Environ. Health Persp..

[31] SCHER Opinion (2015). The Safety of Dental Amalgam and Alternative Dental Restoration Materials for Patients and Users.

[32] Libonati A., Marzo G., Klinger F.G., Farini D., Gallusi G., Tecco S., Mummolo S., De Felici M., Campanella V. (2011). Embryotoxicity assays for leached components from dental restorative materials. Rep. Biol. Endocrinol..

[33] Higgins J.P.T., Green S. (2006). Cochrane Handbook for Systematic Reviews of Interventions.

[34] Moher D., Liberati A., Tetzlaff J., Altman D.G. (2009). PRISMA Group, Preferred reporting items for systematic reviews and meta-analyses: The PRISMA statement. Ann. Int. Med..

[35] Higgins J.P.T., Altman D.G., Gøtzsche P.C., Jüni P., Moher D., Oxman A.D., Savović J., Schulz K.F., Weeks L., Sterne J.A.C. (2011). The Cochrane Collaboration’s tools for assessing risk of bias in randomised trials. BMJ.

[36] Bellinger D.C., Trachtenberg F., Barregard L., Tavares M., Cernichiari E., Daniel D., McKinlay S. (2006). Neuropsychological and Renal Effects of Dental Amalgam in Children. J. Am. Med. Assoc..

[37] Bellinger D.C., Trachtenberg F., Daniel D., Zhang A., Tavares M.A., McKinlay S. (2007). A dose-effect analysis of children’s exposure to dental amalgam and neuropsychological function the New England Children’s Amalgam Trial. J. Am. Dent. Assoc..

[38] Bellinger D.C., Trachtenberg F., Zhang A., Tavares M., Daniel D., McKinlay S. (2008). Dental amalgam and psychosocial status: The New England Children’s Amalgam Trial. J. Dent. Res..

[39] Maserejian N.N., Hauser R., Tavares M., Trachtenberg F.L., Shrader P., McKinlay S. (2012). Dental composites and amalgam and physical development in children. J. Dent. Res..

[40] Shenker B.J., Maserejian N.N., Zhang A., McKinley S. (2008). Immune function effects of dental amalgam in children: A randomized clinical trial. J. Am. Dent. Assoc..

[41] DeRouen T.A., Martin M.D., Leroux B.G., Townes B.D., Woods J.S., Leitão J., Martins I.P. (2006). Neurobehavioral Effects of Dental Amalgam in Children. J. Am. Med. Assoc..

[42] Roberts M.C., Leroux B.G., Sampson J., Luis H.S., Bernardo M., Leitão J. (2008). Dental Amalgam and Antibiotic- and / or Mercury-resistant Bacteria. J. Dent. Res..

[43] Lauterbach M., Martins I.P., Castro-Caldas A., Bernardo M., Luis H., Amaral H., DeRouen T. (2008). Neurological outcomes in children with and without amalgam-related mercury exposure. J. Am. Dent. Assoc..

[44] Celik N., Askın S., Gul M.A., Seven N. (2017). The effect of restorative materials on cytokines in gingival crevicular fluid. Arch. Oral Biol..

[45] Patini R., Spagnuolo G., Guglielmi F., Staderini E., Simeone M., Camodeca A., Gallenzi P. (2020). Clinical Effects of Mercury in Conservative Dentistry: A Systematic Review, Meta-Analysis, and Trial Sequential Analysis of Randomized Controlled Trials. Int. J. Dent..

